# Genetic Association of Toll-Like Receptor 4 (TLR4) Gene Polymorphism (rs4986790) With Oral Squamous Cell Carcinoma (OSCC): A Pilot Case-Control Study

**DOI:** 10.7759/cureus.56021

**Published:** 2024-03-12

**Authors:** Britina Gautam, Anitha Pandi, A. S. Smiline Girija, Paramasivam Arumugam, Vijayashree J Priyadharsini

**Affiliations:** 1 Department of Dentistry, Saveetha Dental College and Hospitals, Saveetha Institute of Medical and Technical Sciences, Saveetha University, Chennai, IND; 2 Centre for Cellular and Molecular Research, Saveetha Dental College and Hospitals, Saveetha Institute of Medical and Technical Sciences, Saveetha University, Chennai, IND; 3 Department of Microbiology, Saveetha Dental College and Hospitals, Saveetha Institute of Medical and Technical Sciences, Saveetha University, Chennai, IND

**Keywords:** polymorphism, toll-like receptor, health, genetic association, genetics, carcinoma

## Abstract

Introduction

Oral squamous cell carcinoma (OSCC) is a highly prevalent and most common form of oral malignancy in the Indian population. Toll-like receptors belong to an important family of receptors that are involved in the process of pathogen recognition and mounting immune response. The expression of this receptor is dysregulated on the tumor cells as reported across several cancer types. The genetic variants in this gene could have a profound impact on the expression of the Toll-like receptor 4 (*TLR4) *gene.

Objective

This study aimed to understand the association of *TLR4* gene polymorphism *(rs4986790) *with OSCC. The objective of this study was to compare the allele and genotype frequencies between the two groups, viz., OSCC and normal healthy subjects, recruited in the study.

Materials and methods

The blood samples were collected from normal healthy subjects (N = 25) and OSCC patients (N = 25). Genomic DNA was isolated from all samples, and genotyping was performed for the *TLR4* gene polymorphism *(rs4986790) *employing the polymerase chain reaction-restriction fragment length polymorphism (PCR-RFLP) approach. The frequency distribution of genotypes and alleles across the study groups was determined by the Chi-square test.

Results

The allele frequency for *TLR4* gene polymorphism *(rs4986790) *in the case group was found to be 60% (A allele) and 40% (G allele), respectively. The study population in both groups were found to agree with the Hardy-Weinberg equilibrium (HWE). The genotype frequency did not differ significantly among the two study groups which was evident from the p-value = 0.8285.

Conclusion

The present study did not report any significant association of the *TLR4* polymorphic marker *rs4986790* with OSCC. Further investigations into the association of other polymorphic markers in the *TLR4* gene, among the larger population of OSCC patients, could provide evidence of their association with OSCC.

## Introduction

Oral squamous cell carcinoma (OSCC) is the most common type of cancer of the oral cavity comprising 80%-90% of all reported neoplasms of the head and neck [1]. A variable incidence pattern has been documented in different nations worldwide. The incidence of OSCC is more pronounced in the South Asian regions, due to exposure to carcinogenic chemicals in tobacco, gutka, pan, etc., More than 0.3 million new cases have been reported to be diagnosed with OSCC in Asia around 2017, and these numbers are expected to increase in the future [2]. Despite improving the therapeutic modalities and exploring new drugs for therapy, the survival of patients remains poor [3]. The malignant phenotype presents with pan-genomic alterations and severely dysregulated protein and gene expression patterns. In recent years, genetic markers have also been identified to have a predominant role to play in the process of oral carcinogenesis [4]. These markers usually fall under the broad category of variations and mutations. These markers are substitutions or changes in the DNA that can markedly affect the transcription and translation of the gene, eventually contributing to alterations in the cellular processes [5]. These variants are of different types such as intron, splice site, missense, nonsense, frameshift, and many more. The variant genotype can confer resistance or susceptibility to a disease [6]. Owing to the steep increase in the number of oral cancer cases in India, there is a pressing need to identify biomarkers with a strong association with the disease. Understanding the nature of these variants, the associated consequences, and their correlation with several clinicopathological features can aid in developing a single-nucleotide polymorphism (SNP) panel that can be employed to diagnose oral cancer at an early stage.

Inflammation is one of the major hallmarks of cancer. The process brings about changes in the inflammatory sites, with the production of pro-inflammatory cytokines, immune cell infiltration, and the interaction between the immune and tissue cells [7]. The inflammatory mediators and their receptors are being explored to demonstrate their role in establishing carcinogenesis [8]. The Toll-like receptor 4 (*TLR4*) gene is associated with innate immunity and has recently been associated with several cancer types. *TLR4* is a transmembrane protein that can initiate myeloid differentiation primary response protein 88 (MyD88)-dependent and independent signaling pathways. They are pattern recognition receptors which are considered to be an important component of the host defense system. The activation of these pathways triggers the cascade responsible for the transcription of inflammatory factors [9]. Recent studies have suggested that the overexpression of Toll-like receptors during infections and inflammatory disease conditions promotes tumorigenesis [10] and immune evasion [11]. The genetic variants in this gene have been found to increase the susceptibility of macular degeneration [12], glaucoma, and preeclampsia [13]. Multiple polymorphisms of the *TLR4* gene in association with various cancer types have been investigated in a meta-analysis conducted by Wang et al. [14]. The A alleles of rs4986790 and rs1927914 and C allele of rs4986791 were found to confer protection against cancer of the prostate, lung, gastric, hepatocellular, and colorectal [14]. A similar kind of meta-analysis reported two SNPs of the *TLR4* gene, viz., rs4986790 and rs4986791, to exhibit an increased risk of cancer with an odd’s ratio of 1.24 [15]. A systematic analysis documented a significant association between the rs4986790 and the tumor. The G allele was considered to increase the risk of gastric cancer, with a strong correlation documented in Asian and Caucasian populations [16]. In addition, the *TLR4* gene expression analysis concerning immune escape and apoptotic resistance to cisplatin in OSCC revealed the fact that TLR4 was overexpressed in OSCC cells, which led to the development of resistance to cisplatin. The team proposed that suppression of TLR4 signaling pathway could improve the cisplatin sensitivity, thereby increasing the survival of patients [17]. 

Investigating these genetic variations in an individual can help the clinician decide on the treatment modalities that can be selected for the patient. The possible outcome of the treatment can also be ascertained by targeting the proteins derived from altered genes [18]. The computational approaches encompassing network/pathway analysis [19], and analysis of gene families, developed through the years have delineated candidate genes associated with cancer [20]. In line with the above observations acquired through an extensive text-mining approach, the present study aimed to reveal the possible association of *TLR4* gene polymorphism (rs4986790) with OSCC in the south Indian population. The aim was achieved by analyzing the genotype and allele frequencies among OSCC patients and normal healthy subjects employing the polymerase chain reaction-restriction fragment length polymorphism (PCR-RFLP) approach and comparing the same with appropriate statistical method.

## Materials and methods

Sample collection

The study was conducted at Saveetha Dental College and Hospitals, Chennai, India. The pilot study included a total of 50 participants divided into two groups, case and control (Table 1). The case group included samples of patients with well-differentiated or moderately differentiated tumors. Well-differentiated tumors presented with extensive keratinization and intercellular bridges, while moderately differentiated tumors presented with focal keratinization. The pilot study was conducted after obtaining institutional human ethical committee approval (No. IHEC/SDC/UG-2014/22/MICRO/223). About 2 mL of venous blood was collected from all the recruited participants. The genomic DNA was isolated from the blood samples following the modified protocol as proposed by Miller et al. [21]. 

**Table 1:** Demographic details and clinical characteristics of the subjects in the OSCC and control groups

Details	OSCC cases (N = 25)	Normal healthy subjects (N = 25)
Participants	Male: 17; female: 8	Male: 17; female: 8
Age range	40-60 years	40-60 years
Smoking habits	N = 17	N = 5
Alcoholic	N = 6	N = 0
Tumor grade	Well-differentiated = 12; moderately differentiated = 13	NA

PCR

**Figure 1:**
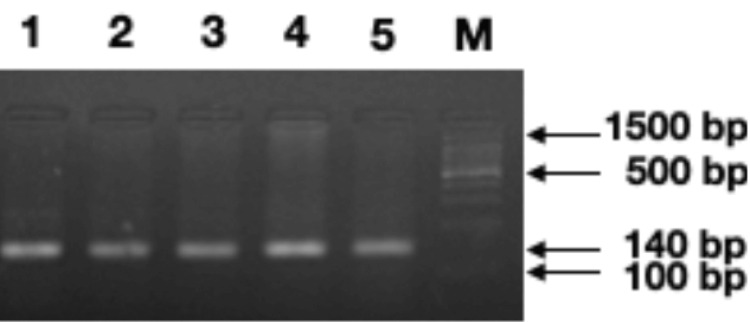
Agarose gel electrophoretogram showing the amplification of the TLR4 gene spanning polymorphic site (rs4986790) running along with standard DNA ladder (lane M=100 bp DNA marker) *TLR4*: Toll-like receptor 4

PCR was conducted to amplify the sequence flanking the polymorphic site in the *TLR4* gene using sequence-specific primers: forward 5’-CTGCTCTAGAGGGCCTGTG-3’ and reverse 5’-TTCAATAGTCACACTCACCAG-3’. The PCR program was set as denaturation at 95°C for 30 sec, annealing at 58°C for 15 sec, and extension at 72°C for 14 sec for 35 cycles. An initial denaturation at 95°C for 5 min and a final extension at 72°C for 5 min were also included. The samples were loaded onto a 2% agarose gel, and a positive amplification signal/product (amplicons) was checked alongside a 100 bp DNA ladder (Takara, Japan) (Figure 1). 

Genotyping Using the RFLP Approach

**Figure 2:**
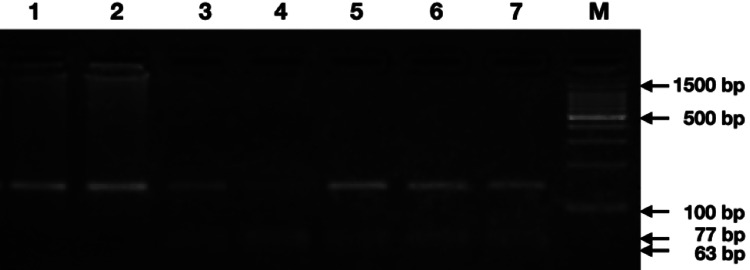
Agarose gel electrophoretogram showing the BccI digested amplicon of TLR4 gene polymorphism (rs4986790) (homozygous wild-type: AA-140 bp (lanes 1 and 2); heterozygous: AG-140 bp + 77 bp + 63 bp (lanes 3, 5-7); homozygous variant: GG-63 bp + 77 bp (lane 4, lane M=100 bp DNA marker) *TLR4*: Toll-like receptor 4

The discrimination between the genotypes was carried out by the RFLP. The amplicons were digested using the BccI enzyme (New England Biolabs, USA) for 2 hrs at 37°C. The BccI-digested product was resolved in a 3% agarose gel. The genotypes were based on the band patterns where the homozygous GG variant produced two bands of size 77 bp + 63 bp, the homozygous wild-type AA variant produced one band of size 140 bp, and the heterozygous GA produced three bands of size 140 bp + 77 bp + 63 bp (Figure 2).

Statistical analysis

The genotype and allele frequencies were compared between the two groups using the Chi-square test, where a p-value less than 0.05 was considered to be significant. The study groups were found to be in agreement with the Hardy-Weinberg equilibrium (HWE). All of the statistical analyses were performed using the IBM SPSS Statistics for Windows, Version 23.0 (Released 2015; IBM Corp., Armonk, New York, United States). The comparison of the allele frequencies acquired from the present study with that of the other population was performed by employing the data derived from the Ensembl database for the SNP marker rs4986790 [22].

## Results

Demographic details

The participants were age- and gender-matched, with a male-to-female ratio of 2:1 (male, N = 17; and female, N = 8) belonging to the same population. The OSCC patients and normal healthy subjects were enrolled in the study after obtaining informed consent. The age range was found to be between 40 and 60 years. Among the OSCC group, 17 patients were smokers and six were found to consume alcohol. On the other hand, five subjects were found to be smokers in the control group. The OSCC patients presented with well and moderately differentiated tumors (Table 1). The normal healthy subjects were found to be free of any oral lesions including potentially malignant conditions. The females who were pregnant or lactating were excluded from the study. The healthy volunteers did not receive any antibiotic treatment before three months of conducting the study.

Genotype analysis

The missense variant, rs4986790 of the *TLR4* gene, leads to a change of the amino acid aspartic acid at the 299th position to a glycine. The A allele was found to be the ancestral allele, and the G allele is a variant. The allele frequencies of the *TLR4* gene polymorphism rs4986790 in the case group were found to be 60% for the A allele and 40% for the G allele. The distribution of genotypes among the OSCC group was found to be 32%, 56%, and 12% for the AA, AG, and GG genotypes, respectively. The frequency of the AA homozygous wild-type genotype was found to be high in the control group (40%) when compared to the case group. In contrast, the frequency of heterozygous genotype AG was found to be high in the case group (56%). The study groups were found to comply with the HWE which is evident from the p-value of 0.4046 for cases and 0.8349 for controls (Table 2). The genotype frequency comparison between the case and control groups produced a p-value of 0.8285. This comparison did not exhibit any statistical significance; hence, it is concluded that there is no association between the *TLR4* gene polymorphism (rs4986790) and OSCC. 

Comparison of population data

**Table 2:** The overall genotype and allele frequency distribution of TLR4 gene polymorphism (rs4986790) among the study groups *TLR4*: Toll-like receptor 4 *For deviation from Hardy-Weinberg equilibrium (HWE), Chi-square with one degree of freedom. The genotype frequency of cases and controls does not differ significantly χ2df (p = 0.8285). A p-value of <0.05 was considered to be significant.

Groups	AA (%)	AG (%)	GG (%)	A	G	HWE* (p-value)
Case (N = 25)	8 (32)	14 (56)	3 (12)	0.60	0.40	0.4046
Control (N = 25)	10 (40)	12 (48)	3 (12)	0.64	0.36	0.8349

**Figure 3:**
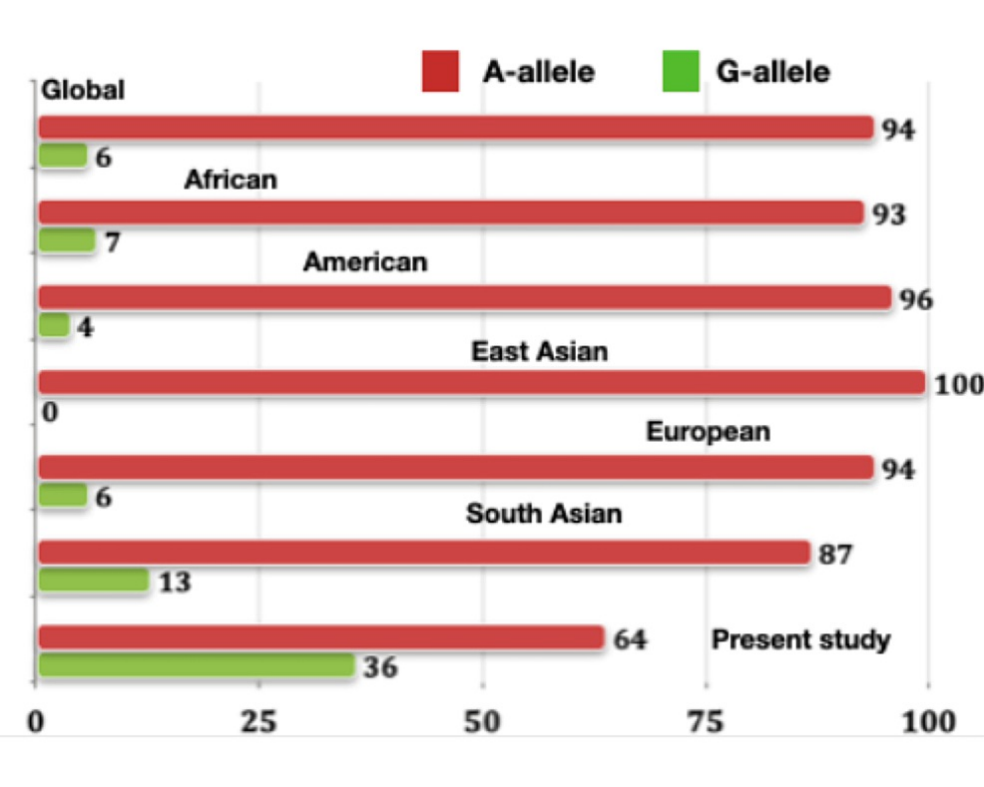
The graph depicts the allele frequency of TLR4 gene polymorphism (rs4986790) in different populations (data acquired from Ensembl database) *TLR4*: Toll-like receptor 4

The comparison of allele frequencies among different populations with the data acquired from the present study was performed with the information retrieved from the Ensembl database (https://asia.ensembl.org/Homo_sapiens/Variation/Population?db=core;r=9:117712524-117713524;v=rs4986790;vdb=variation;vf=730249947). The data was accessed on September 24, 2023. All data presented in the Figure 3 represents the allele frequency values presented as percentage. The present study demonstrated the highest frequency of G allele (36%) in the control group (N = 25). In contrast, the global population presented with 94% of the A allele and 6% of the G allele. The East Asian population showed a monomorphic state with the predominance of the A allele, while the South Asian population presented with 13% frequency of the variant allele G (Figure 3). The results of the present study are comparable to the South Indian population data; however, information from a larger dataset is required to prove the fact [22].

## Discussion

The study of the genetic association between *TLR4* gene polymorphism (rs4986790) and OSCC holds great significance in understanding the genetic factors that influence the susceptibility of OSCC. The knowledge obtained from this study can help in developing personalized prevention and treatment strategies, which can result in early detection and intervention for individuals at higher risk of OSCC. A review conducted with over 20 years of data on the role of Toll-like receptors confirmed that *TLR2*, *3*, *4*, *5*, *7*, and *9* are associated with the development and progression of OSCC [23]. Numerous single-nucleotide variants and mutations were identified in the *TLR4* gene with strong association with several cancer types. A study conducted by Priyadarshini et al., on the association of Asp299Gly and Thr399Ile polymorphism of *TLR4* gene in the North Indian population, demonstrated a high prevalence of AG genotype (16.6%) in prostate cancer patients (N = 198) [24]. They also showed that the AA and the AG genotypes were protective and risk genotypes, as evidenced by an odd’s ratio of 0.39 and 4.4, respectively [24]. A similar observation was made in patients with ovarian cancer (OC). Around 200 female subjects were recruited from various healthcare centers in Poland, of which 70 were diagnosed with OC. The investigators conducted genotyping for the rs4986790 polymorphism. They reported the highest frequency of heterozygous genotype and minor alleles of the polymorphism, which were AG and G, respectively. They concluded that the Asp299Gly variation could increase the risk of developing OC [25]. 

A study among 100 Tunisian patients diagnosed with colorectal cancer provided a significant association with stage and lymph node metastasis. However, they could not prove a significant association of D299G polymorphism with colorectal cancer susceptibility [26]. The association of *TLR4* polymorphism (rs4968790) with clinical and pathological features in melanoma patients was assessed. The study population included 120 patients, and the genotyping was carried out employing TaqMan real-time PCR assay. The *TLR4* D299G and T399I variants were associated with nodal metastasis, advanced stage III, and severity of melanoma [27]. A very recent study by Jha and colleagues demonstrated that the AG genotype and the G allele were found to be the risk genotype and risk allele in the population studied. They assessed several SNPs of *TLR4 *and *TLR9* genes with respect to different phenotypes such as bacterial infection, gingival inflammation/recession, and OSCC. They employed techniques such as RT-PCR, Western blot analysis, allele-specific PCR, and sequencing to investigate the same [28]. The present study was an attempt to provide an answer to the research question about the association between the *TLR4* gene polymorphism (rs4986790) and the susceptibility to OSCC and how this genetic variant contributes to the risk and progression of OSCC. The study results demonstrated a close agreement with the other reports, reiterating the fact that the heterozygous genotype AG could be the oral cancer case when compared to normal healthy subjects. Another finding by de Barros Gallo et al. screened for the rs4696480 and other TLR polymorphisms in OSCC and potentially malignant oral disorders. They also included lung squamous cell carcinoma samples in the study [29]. This study showed that the A allele was the risk allele in the Spanish population studied. This report was contrary to that which was documented in other populations. These results could be attributed to the population, habits, and other exposures contributing to the disease phenotype [29]. Therefore, a population-wide study might help in delineating the genetic markers unique to the population.

Limitations

The limitations of the present study were (a) the pilot study recruited a small sample size of 25 individuals in each of the two groups. The study could be expanded to include a larger sample size; (b) only one polymorphic marker was selected based on an extensive text-mining approach; however, there are numerous other polymorphisms in the *TLR4* gene that could show a positive association with OSCC; and (c) the study results were not stratified based on age, gender, or tumor grade owing to a small sample size. 

## Conclusions

The present study reported a relatively high frequency of heterozygous genotype among oral cancer patients. Further studies on larger sample sizes, different ethnic groups, exposures, and other factors would provide concrete evidence about the role of *TLR4* gene variants in establishing a cancer phenotype. The future prospects of SNP analysis in complex disorders like cancer would open new avenues in precision or personalized medicine as the cumulative effect of multiple deleterious or pathogenic variants can have a profound impact on the disease phenotype. A whole-genome or exome analysis would aid us in selecting a hub of candidate polymorphic markers that can be used for screening and designing therapeutic leads for oral cancer.

## References

[1] Johnson NW, Jayasekara P, Amarasinghe AA (2011). Squamous cell carcinoma and precursor lesions of the oral cavity: epidemiology and aetiology. Periodontol 2000.

[2] Kashyap B, Mikkonen JJ, Bhardwaj T, Dekker H, Schulten EA, Bloemena E, Kullaa AM (2022). Effect of smoking on MUC1 expression in oral epithelial dysplasia, oral cancer, and irradiated oral epithelium. Arch Oral Biol.

[3] Abijeth B, Ezhilarasan D (2020). Syringic acid induces apoptosis in human oral squamous carcinoma cells through mitochondrial pathway. J Oral Maxillofac Pathol.

[4] Eljabo N, Nikolic N, Carkic J, Jelovac D, Lazarevic M, Tanic N, Milasin J (2018). Genetic and epigenetic alterations in the tumour, tumour margins, and normal buccal mucosa of patients with oral cancer. Int J Oral Maxillofac Surg.

[5] Alzu'bi AA, Zhou L, Watzlaf VJ (2019). Genetic variations and precision medicine. Perspect Health Inf Manag.

[6] Deng N, Zhou H, Fan H, Yuan Y (2017). Single nucleotide polymorphisms and cancer susceptibility. Oncotarget.

[7] Colotta F, Allavena P, Sica A, Garlanda C, Mantovani A (2009). Cancer-related inflammation, the seventh hallmark of cancer: links to genetic instability. Carcinogenesis.

[8] Korneev KV, Atretkhany KN, Drutskaya MS, Grivennikov SI, Kuprash DV, Nedospasov SA (2017). TLR-signaling and proinflammatory cytokines as drivers of tumorigenesis. Cytokine.

[9] Chen X, Zhao F, Zhang H, Zhu Y, Wu K, Tan G (2015). Significance of TLR4/MyD88 expression in breast cancer. Int J Clin Exp Pathol.

[10] Li J, Yang F, Wei F, Ren X (2017). The role of toll-like receptor 4 in tumor microenvironment. Oncotarget.

[11] Tang X, Zhu Y (2012). TLR4 signaling promotes immune escape of human colon cancer cells by inducing immunosuppressive cytokines and apoptosis resistance. Oncol Res.

[12] Liu XC, Guo XH, Chen X, Yao Y (2020). Toll-like receptor 4 gene polymorphisms rs4986790 and rs4986791 and age-related macular degeneration susceptibility: a meta-analysis. Ophthalmic Genet.

[13] Sun M, Jiang H, Meng T, Liu P, Chen H (2021). Association between TLR4 gene polymorphisms and risk of preeclampsia: systematic review and meta-analysis. Med Sci Monit.

[14] Wang F, Wen X, Wen T, Liu Z (2023). Association of TLR4 gene 2026A/G (rs1927914), 896A/G (rs4986790), and 1196C/T (rs4986791) polymorphisms and cancer susceptibility: meta-analysis and trial sequential analysis. Medicine (Baltimore).

[15] Zhang K, Zhou B, Wang Y, Rao L, Zhang L (2013). The TLR4 gene polymorphisms and susceptibility to cancer: a systematic review and meta-analysis. Eur J Cancer.

[16] Xiao Q, Chen J, Zeng S, Cai H, Zhu G (2022). An updated systematic review of the association between the TLR4 polymorphism rs4986790 and cancers risk. Medicine (Baltimore).

[17] Sun Z, Luo Q, Ye D, Chen W, Chen F (2012). Role of toll-like receptor 4 on the immune escape of human oral squamous cell carcinoma and resistance of cisplatin-induced apoptosis. Mol Cancer.

[18] Jayaseelan VP, Ramesh A, Arumugam P (2021). Breast cancer and DDT: putative interactions, associated gene alterations, and molecular pathways. Environ Sci Pollut Res Int.

[19] Aditya J, Smiline Girija AS, Paramasivam A, Vijayashree Priyadharsini J (2021). Genetic alterations in Wnt family of genes and their putative association with head and neck squamous cell carcinoma. Genomics Inform.

[20] Devi SK, Paramasivam A, Girija AS, Priyadharsini JV (2021). Decoding the genetic alterations In cytochrome P450 family 3 genes and its association with HNSCC. Gulf J Oncolog.

[21] Miller SA, Dykes DD, Polesky HF (1988). A simple salting out procedure for extracting DNA from human nucleated cells. Nucleic Acids Res.

[22] Kersey PJ, Allen JE, Christensen M (2014). Ensembl Genomes 2013: scaling up access to genome-wide data. Nucleic Acids Res.

[23] Sharma Y, Bala K (2020). Role of Toll like receptor in progression and suppression of oral squamous cell carcinoma. Oncol Rev.

[24] Priyadarshini A, Chakraborti A, Mandal AK, Singh SK (2013). Asp299Gly and Thr399Ile polymorphism of TLR-4 gene in patients with prostate cancer from North India. Indian J Urol.

[25] Kania KD, Haręża D, Wilczyński JR, Wilczyński M, Jarych D, Malinowski A, Paradowska E (2022). The Toll-like receptor 4 polymorphism Asp299Gly is associated with an increased risk of ovarian cancer. Cells.

[26] Omrane I, Baroudi O, Kourda N (2014). Positive link between variant Toll-like receptor 4 (Asp299Gly and Thr399Ile) and colorectal cancer patients with advanced stage and lymph node metastasis. Tumour Biol.

[27] Ostojic N, Radevic T, Kandolf Sekulovic L, Djordjevic B, Jaukovic L, Stepic N, Supic G (2022). Polymorphisms in toll-like receptor 3 and 4 genes as prognostic and outcome biomarkers in melanoma patients. Melanoma Res.

[28] Jha A, Nath N, Kumari A, Kumari N, Panda AK, Mishra R (2023). Polymorphisms and haplotypes of TLR-4/9 associated with bacterial infection, gingival inflammation/recession and oral cancer. Pathol Res Pract.

[29] de Barros Gallo C, Marichalar-Mendia X, Setien-Olarra A (2017). Toll-like receptor 2 rs4696480 polymorphism and risk of oral cancer and oral potentially malignant disorder. Arch Oral Biol.

